# Vector Transmission of Tomato Yellow Leaf Curl Thailand Virus by the Whitefly *Bemisia tabaci*: Circulative or Propagative?

**DOI:** 10.3390/insects12020181

**Published:** 2021-02-20

**Authors:** Wei-Hua Li, De-Fen Mou, Chien-Kuei Hsieh, Sung-Hsia Weng, Wen-Shi Tsai, Chi-Wei Tsai

**Affiliations:** 1 Department of Entomology, National Taiwan University, Taipei 10617, Taiwan; f06632007@ntu.edu.tw (W.-H.L.); defenmou@ufl.edu (D.-F.M.); r02632008@ntu.edu.tw (C.-K.H.); kgket4007@gmail.com (S.-H.W.); 2 Department of Plant Medicine, National Chiayi University, Chiayi 600355, Taiwan; wenshi.tw@mail.ncyu.edu.tw

**Keywords:** *Begomovirus*, *Bemisia tabaci*, transovarial transmission, transmission mode, virus replication

## Abstract

**Simple Summary:**

Tomato yellow leaf curl viruses cause disease epidemics in tomato crops in tropical and subtropical areas worldwide. The sweet potato whitefly, *Bemisia tabaci*, is a vector of this group of viruses. This research studied the transmission biology of tomato yellow leaf curl Thailand virus (TYLCTHV) by *B. tabaci*, including virus-infected tissues, virus translocation, virus replication, and transovarial transmission (i.e., transmission from mother to progeny via ovaries). We discovered that the virus first infects the alimentary gut, then the hemolymph, and finally the salivary glands of the whitefly. The virus did not replicate in the whitefly during infection. In addition, TYLCTHV was detected in only 10% of infected females’ first-generation progeny, but the progeny was unable to cause viral infection of tomato plants; therefore, there was no evidence of transovarial transmission. When combined with the current literature, our results suggest that *B. tabaci* transmits TYLCTHV in a persistent-circulative mode.

**Abstract:**

Viruses that cause tomato yellow leaf curl disease are part of a group of viruses of the genus *Begomovirus*, family *Geminiviridae*. Tomato-infecting begomoviruses cause epidemics in tomato crops in tropical, subtropical, and Mediterranean climates, and they are exclusively transmitted by *Bemisia tabaci* in the field. The objective of the present study was to examine the transmission biology of the tomato yellow leaf curl Thailand virus (TYLCTHV) by *B. tabaci*, including virus-infected tissues, virus translocation, virus replication, and transovarial transmission. The results demonstrated that the virus translocates from the alimentary gut to the salivary glands via the hemolymph, without apparent replication when acquired by *B. tabaci*. Furthermore, the virus was detected in 10% of the first-generation progeny of viruliferous females, but the progeny was unable to cause the viral infection of host plants. There was no evidence of transovarial transmission of TYLCTHV in *B. tabaci*. When combined with the current literature, our results suggest that *B. tabaci* transmits TYLCTHV in a persistent-circulative mode. The present study enhances our understanding of virus–vector interaction and the transmission biology of TYLCTHV in *B. tabaci*.

## 1. Introduction

A majority of pathogenic plant viruses are known to have associations with insect vectors, which are responsible for virus transmission among plant hosts [1,2]. Especially, the persistently transmitted plant viruses rely exclusively on insect vectors for their spread in the field [3]. Many insect-transmitted, crop-infecting viruses cause significant losses in crop yields [4,5,6]. Therefore, the study of virus–vector interaction, including acquisition, translocation, and accumulation of a virus within an insect vector, is important for fully understanding virus transmission and epidemiology [7,8]. Ultimately, this will contribute to the development of disease-management programs.

Tomato yellow leaf curl viruses comprise a group of viruses of the genus *Begomovirus* in the family *Geminiviridae* [9]. They are exclusively transmitted by the whitefly *Bemisia tabaci* [5]. Tomato-infecting begomoviruses cause epidemics in tomato crops in tropical, subtropical, and Mediterranean climates [6,10,11]. Among them, tomato yellow leaf curl virus (TYLCV) was first discovered in Israel [12], whilst tomato yellow leaf curl Thailand virus (TYLCTHV) is the predominant virus species in the tomato fields of Taiwan [13], and it is also present in Thailand and Yunnan, China [14,15]. TYLCTHV has caused great economic damage to tomato production in Thailand [16].

It has been suggested that *B. tabaci* transmits TYLCV in a persistent-circulative mode [17], which means that the virus translocates from the alimentary gut to the salivary glands via the hemolymph, without replicating when acquired by *B. tabaci*. Some studies support this idea [18,19], whilst others have demonstrated that TYLCV both circulates in the hemolymph and replicates in *B. tabaci* [20,21,22,23]. Therefore, whether *B. tabaci* transmits tomato-infecting begomoviruses in a persistent-circulative or persistent-propagative manner is still a controversial issue.

In addition to replicating in insect vectors, TYLCV has shown vertical transmission in viruliferous female whiteflies [24,25,26,27], referred to herein as “transovarial passage”. Among these studies, some have demonstrated that the virus can be detected in the progeny but is unable to cause host-plant infection [25,26]. In contrast, Ghanim et al. [24] and Wei et al. [27] reported that *B. tabaci* that had inherited TYLCV from a viruliferous mother could cause viral infection of host plants (i.e., transovarial transmission). So far, transovarial transmission has only been evidenced in TYLCV but not in other tomato begomoviruses.

Control programs for insect-transmitted plant diseases need to be based on a solid understanding of the causative pathogen’s transmission biology in order to be effective. However, at present, the virus–vector interaction and transmission biology of TYLCTHV remain unclear. The objective of the present study was to examine the transmission biology of TYLCTHV in *B. tabaci*, including virus-infected tissues, virus translocation, virus replication, and transovarial transmission. Our results provide important insights into the transmission mechanism of TYLCTHV by *B. tabaci*.

## 2. Materials and Methods

### 2.1. Insect, Virus, and Plants

The sources of our *B. tabaci* colony and TYLCTHV are described in Weng et al. [28]. The laboratory colony of *B. tabaci* Middle East–Asia Minor 1 (MEAM1) putative species was reared on Chinese kale (*Brassica oleracea* cv. Alboglabra Group) in whitefly-proof net cages (30 × 30 × 30 cm^3^, Megaview, Taichung, Taiwan), at 28 °C, under a photoperiod of L:D 16:8 h. Chinese kale is a non-host of TYLCTHV; therefore, whiteflies reared on it are non-viruliferous.

TYLCTHV was maintained in tomato plants (*Solanum lycopersicum* cv. ANT22), by vector transmission, using *B. tabaci*. TYLCTHV-infected and healthy tomato plants were prepared and maintained as previously described [29]. Chinese kale and tomato plants were grown from seeds and cultured in an environmental growth chamber, under the aforementioned conditions. Tomato seedlings with 3–5 true leaves were used as healthy test plants for insect-transmission experiments.

### 2.2. Virus-Infected Tissues of B. tabaci

The accumulation of TYLCTHV in *B. tabaci* was examined with an immunofluorescence assay modified from Guo et al. [30]. Non-viruliferous adult whiteflies (0–5 days old) were transferred, by mouth aspirator, to the aforementioned net cage enclosed with TYLCTHV-infected tomato plants and allowed 5-day acquisition access periods (AAP). Viruliferous whiteflies were then dissected in 0.01 M phosphate-buffered saline (PBS, pH 7.4), under a stereomicroscope. Six legs were pulled off in order to collect the hemolymph, which was then transferred to a silane-coated microscope slide (Muto pure chemicals, Tokyo, Japan), so that the hemocytes could adhere. The recovered organs (midgut, hindgut, primary salivary gland (PSGs), and ovariole) and hemocytes on the silane-coated slide were then treated with 4% paraformaldehyde, at 25 °C, for 30 min; incubated with PBS containing 0.1% Triton-X 100 (PBST), at 4 °C, overnight; and then blocked with 1% bovine serum albumin (BSA), in PBS, at 25 °C, for 1 h. Next, the specimens were incubated with TYLCTHV antibody (200× dilution in PBST with 0.1% BSA), for 1.5 h, followed by incubation in goat anti-rabbit immunoglobulin G (IgG) antiserum conjugated with Alexa Fluor 555 (200× dilution, Invitrogen, Carlsbad, CA, USA), for 1 h, at room temperature. TYLCTHV antibody (AS-0952) was kindly provided by Dr. Wulf Menzel (Leibniz Institute DSMZ-GmbH, Braunschweig, Germany). Finally, the specimens were rinsed with PBST three times, to remove any non-specific binding and mounted in the SlowFade Gold Antifade Mountant with 4’-6-diamidino-2-phenylindole dihydrochloride (DAPI) (Invitrogen). The specimens were then examined with a Leica TCS SP5 II confocal laser-scanning microscope. Each organ had 12 biological replicates.

### 2.3. Virus Translocation in B. tabaci

To investigate virus translocation after *B. tabaci* acquiring TYLCTHV, the presence of the virus in *B. tabaci* organs after various AAPs was examined by PCR assays. Approximately 20 adults (0–5 days old) were transferred to a detached leaf of a TYLCTHV-infected tomato plant and placed in a plastic cup (250 mL) for 2, 3, 4, 14, or 24 h of feeding. The petiole of the detached leaf was inserted into a glass vial of water, to prevent wilting. After the acquisition period, the whiteflies were transferred to a Chinese kale leaf for 2 h, to clear possible gut residue from the infected phloem sap. At each time point, 10 whiteflies were collected, and their midgut, PSGs, and legs (a surrogate for hemolymph) were removed. DNA was extracted from the recovered organs, using QuickExtraction DNA Extraction Solution (Epicentre, Madison, WI, USA) as previously described [28]. PCR was performed, using recombinant *Taq* DNA polymerase (Invitrogen) and the TYLCTHV-specific primer set ThCP-F (5’-ACGCCCGTCTCGAAAGTA-3’) and ThCP-R (5’-CCAGCTTCCTGGTGATTGTA-3’) [29]. PCR conditions were as follows: initial denaturation (94 °C, 3 min); 40 cycles of denaturation (94 °C, 45 s), annealing (52 °C, 30 s), and extension (72 °C, 1 min); and a final extension (72 °C, 10 min). Ten of each organ at each time point were tested.

### 2.4. Time-Series Quantification of the Virus in B. tabaci

To determine whether TYLCTHV replicates in *B. tabaci*, the viral genome in viruliferous whitefly was quantified by real-time PCR, after the whitefly had fed on Chinese kale. Approximately 300 adult whiteflies (0–5 days old) were transferred by mouth aspirator to a net cage enclosed with a TYLCTHV-infected tomato plant for 24 h of feeding. Subsequently, the whiteflies were transferred to another net cage enclosed with a Chinese kale. Ten females and 10 males were then randomly collected by mouth aspirator every 24 h until day 10. Each pool of 10 whiteflies was stored in a 1.7 mL microcentrifuge tube, at −80 °C, until DNA extraction.

The DNA of each whitefly pool was extracted, using DNeasy Blood and Tissue kit (Qiagen, Valencia, CA, USA), following the manufacturer’s instructions. The TYLCTHV genome was quantified by real-time PCR, as previously described [29], using the TYLCTHV-specific primer set THqAV2-F (5’-AAGTATCTGCAAGCGGTCGAGAA-3’) and THqAV2-R (5’-ACGGGGGTGGAAATGAGAAT-3’) [29]. Part of the *heat shock protein 90* (*HSP90*) gene of *B. tabaci* was amplified, using the primer set HSP90-F (5’-ATCGCCAAATCTGGAACTAAAGC-3’) and HSP90-R (5’-GTGTTTTGAGACGACT GTGACGGTG-3’) [31], to serve as an internal control. Both primer sets were validated with a PCR efficiency of 100% and 98%, respectively. Melting curves were generated at the end of each reaction, to confirm the specificity of the amplification. Experiments were repeated 6 times. The quantity of TYLCTHV genome relative to the *HSP90* gene was calculated, using the 2^−∆Ct^ method.

Relative quantities of TYLCTHV genome between days were analyzed, using the Kruskal–Wallis test. All data analyses were performed, using SPSS 22.0.

### 2.5. Transovarial Passage and Transmission

Twenty female and 20 male adult whiteflies (0–2 days old) were given an AAP of 48 h on a TYLCTHV-infected tomato plant in the aforementioned net cage. After the AAP, all viruliferous whiteflies were transferred, by mouth aspirator, to another net cage and enclosed with a Chinese kale for an additional 5 days for oviposition. During this period, females had the opportunity to lay eggs on the Chinese kale. All adults were removed from the Chinese kale on the sixth day, with only the eggs and nymphs of first-generation progeny (F1) remaining on the plant. In this setting, the progeny of the viruliferous whiteflies hatched and developed on the Chinese kale, without contact of TYLCTHV. Twenty-five of the F1 progeny were individually subjected to DNA extraction and PCR assay when they emerged as adults, to examine whether they had inherited TYLCTHV from their viruliferous mothers. Additionally, to evaluate whether the transovarially transmitted TYLCTHV in the F1 progeny was infectious, 5 adults of the F1 progeny were used to inoculate healthy tomato plants by enclosing them with a tomato leaf in a gauze bag (6 × 15 cm^2^, 109 mesh/inch, Megaview) for an inoculation access period (IAP) of 48 h. Twenty-five test plants were inoculated by the F1 progeny. After the IAP, inoculated plants were treated with a systemic insecticide (acetamiprid), to kill all remaining insects on the plants. PCR assays were conducted to assess the infection of TYLCTHV in the inoculated plants three weeks after the IAP. DNA extraction from tomato leaves and whiteflies, as well as the PCR procedure, was performed as described previously [28]. Four replicates were conducted for this experiment. A total of 100 of the F1 progeny were tested, and 100 test plants were inoculated with the F1 progeny and examined. Negative control experiments were carried out, using the same batch of test plants that had not been exposed to viruliferous *B. tabaci*, and were kept in the same environmental growth chamber as the inoculated test plants, until the PCR assay.

## 3. Results

### 3.1. Virus-Infected Tissues of B. tabaci

**Figure 1:**
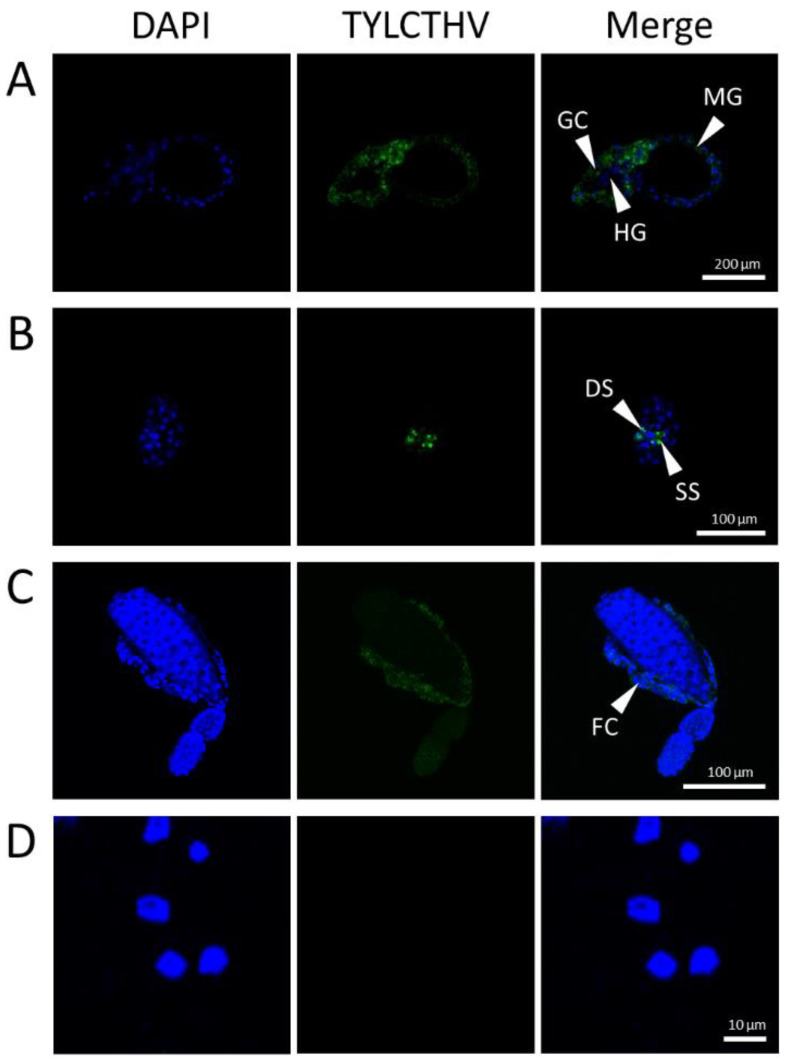
TYLCTHV accumulation (green color) in (**A**) alimentary gut, (**B**) primary salivary gland, (**C**) ovariole, and (**D**) hemocytes of viruliferous *B. tabaci*. Specimens were probed successively with TYLCTHV antibody and Alexa Fluor 555 conjugated secondary antibody. The nuclei of cells were stained with DNA-selective dye DAPI (blue color). Abbreviations: DS, ductal section; FC, follicle cells; GC, gastric caeca; HG, hindgut; MG, midgut; SS, secretory section.

An immunofluorescence assay was conducted to detect the accumulation of TYLCTHV in *B. tabaci*. For the alimentary gut, an Alexa Fluor 555 signal was detected in the midgut, gastric caeca, and filter chamber, but not in the hindgut of viruliferous whiteflies, indicating that TYLCTHV accumulated in the midgut, gastric caeca, and filter chamber of *B. tabaci* (Figure 1A). A fluorescence signal was also observed in the PSGs of viruliferous whiteflies; strong signals were observed in the secretory and ductal sections, whereas the endcaps displayed weaker signals (Figure 1B). Among the organs that were positive for TYLCTHV accumulation, the midgut, gastric caeca, filter chamber, ovarioles, and PSGs showed infection rates of 100% (Table 1). For ovarioles, a signal was only detected in follicle cells of viruliferous whiteflies, but not in the oocyte (Figure 1C). No signal was detected in the hemocyte of viruliferous whiteflies (Figure 1D). All negative controls (i.e., non-viruliferous whiteflies) showed no Alexa Fluor 555 signal (data not shown), indicating that no TYLCTHV accumulated in them.

### 3.2. Virus Translocation in B. tabaci

A time-series PCR assay was conducted to investigate virus translocation in *B. tabaci* after acquiring TYLCTHV. The midgut, hemolymph, and PSGs were examined by PCR for the presence of TYLCTHV at 2, 3, 4, 14, and 24 h after the start of AAP. None of the organs tested positive for TYLCTHV at 2 h (Table 2), and the virus was first detected in the midgut and hemolymph at 3 h. The TYLCTHV infection rate of the midgut reached 100% 14 h after the start of the AAP. At the same time, the infection rates of the hemolymph and PSGs were 70% and 80%, respectively (Table 2). These results demonstrate that TYLCTHV first infects the midgut, followed by the hemolymph and then PSGs of *B. tabaci*.

### 3.3. Virus Replication in B. tabaci

**Figure 2:**
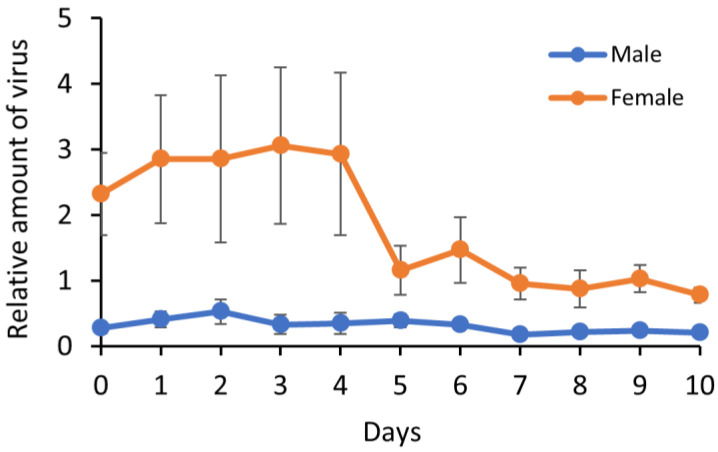
Relative amount of TYLCTHV in viruliferous *B. tabaci*. The virus was quantified by real-time PCR after an acquisition access period of 24 h and then transferred to Chinese kale, a non-host of the virus. The amount of virus was normalized to the *HSP90* gene of *B. tabaci*. Vertical bars represent standard error of the mean (*n* = 6).

A time-series real-time PCR assay was conducted, to investigate whether TYLCTHV replicates in *B. tabaci*. The viral load in females remained constant during the first four days and then decreased to low levels, from day 5 to day 10 (Figure 2, Table S1); however, there were no significant differences between days (Kruskal–Wallis test, *p* = 0.36). Regression analysis showed that the relationship between viral load and time was negatively correlated with a slope of −2.47, suggesting that viral load decreased with time. However, in males, the viral load did not significantly alter over the course of 10 days (Figure 2; Kruskal–Wallis test, *p* = 0.56). The results therefore suggest that TYLCTHV does not replicate in viruliferous *B. tabaci*.

### 3.4. Transovarial Passage and Transmission

To determine whether TYLCTHV is transovarially transmitted by *B. tabaci*, the F1 progeny of viruliferous females were examined by PCR and a transmission assay. Ten F1 individuals tested positive for TYLCTHV from a total of 100. However, none of the tomato plants (*n* = 100) inoculated with F1 progenies tested positive for TYLCTHV. None of the negative controls tested positive for TYLCTHV. The results imply that TYLCTHV is inherited from viruliferous mother to F1 progeny, with 10% of F1 adults carrying the virus; however, the F1 progeny is unable to cause viral infection of host plants.

## 4. Discussion

The confocal microscopy results demonstrate that TYLCTHV accumulates in the PSGs, gastric caeca, midgut, and filter chamber of the *B. tabaci* alimentary gut. This is similar to virus accumulation in *B. tabaci* reported for TYLCV, tomato yellow leaf curl China virus (TYLCCNV), and tomato yellow leaf curl Sardinia virus (TYLCSV) [32,33,34,35]. The TYLCTHV viral load was found to be heavier in the secretory and ductal sections of PSGs than in the endcaps. These results imply that TYLCTHV translocates from the PSGs to saliva. The secretory section of the PSG is critical in vector specificity. For example, TYLCCNV penetrates and accumulates in the secretory section of PSGs in *B. tabaci* MEAM1 species but not Mediterranean (MED) species, whereas TYLCV can be detected in the secretory section of PSGs of both MEAM1 and MED species [35].

Furthermore, we found that TYLCTHV accumulated in the ovarioles but not oocytes of *B. tabaci*, through confocal microscopy. A fluorescence signal was only detected in follicle cells surrounding the oocyte. However, 10% of the F1 progeny harbored the virus, as confirmed by PCR. Therefore, it is likely that the F1 progeny of viruliferous females inherit TYLCTHV, but the amount of virus in most F1 progeny is below the PCR detection threshold. Their viral loads were also too low to be detected by the immunofluorescence assay.

The F1 progeny of viruliferous females cannot cause viral infection of host plant; however, some progeny inherit TYLCTHV from viruliferous females. Transovarial passage is possible in the TYLCTHV-*B. tabaci* MEAM1 pathosystem, but the transovarial transmission of TYLCTHV was not supported by our data. Our results are similar to those reported in the transmission of TYLCSV by *B. tabaci* [36], where the authors suggested that the DNA of TYLCSV, but not entire virus particles, was inherited by F1 progeny. Two potential explanations for why none of the susceptible plants inoculated by F1 progeny tested positive for TYLCTHV are as follow: (1) F1 progeny do not inherit entire virus particles, and (2) a higher amount of TYLCTHV is required for *B. tabaci* MEAM1 species to transmit the virus to plants. So far, transovarial transmission has only been evidenced in TYLCV, but not in other tomato begomoviruses [24,27]. However, not all species of the *B. tabaci* complex transmit TYLCV transovarially. Guo et al. [37] tested the possibility of the transovarial transmission of TYLCV by seven *B. tabaci* species and discovered that this only occurred in MEAM1 species.

Whether tomato begomoviruses replicate in whitefly vectors is still controversial. Some studies have reported that the amount of TYLCV in *B. tabaci* increases with time after viruliferous whiteflies are transferred to a non-host plant of the virus [20,21]; other studies report that the amount increases in the first two days and then gradually decreases [22,23]. However, it has also been reported that neither TYLCV [18,19,34] nor TYLCSV [38] replicates in *B. tabaci*. In the present study, the amount of TYLCTHV in female whiteflies gradually decreased with time, whilst remaining constant in males.

The present TYLCTHV translocation study demonstrated that the virus infected the midgut and hemolymph within 3 h, and then the PSGs within 14 h of feeding on an infected plant. The time needed for TYLCTHV to infect the PSGs is useful for disease control, since the virus cannot be transmitted to other host plants until it reaches the PSGs. The transmission characteristics of TYLCTHV by *B. tabaci* MEAM1 species were previously examined by our group [28], and illustrated that the minimum acquisition time is 2 h, retention time is lifelong, and latent period is less than 8 h [28]. According to these characteristics (acquisition time in hours, hours of latent period, lifelong retention, virus in hemolymph, no replication in vector, and no transovarial transmission), we conclude that the transmission mode of TYLCTHV is persistence-circulative [3].

## 5. Conclusions

TYLCTHV infects and accumulates in the PSGs, gastric caeca, midgut, and filter chamber of *B. tabaci* MEAM1 species, but not the hindgut and oocyte. When acquired by *B. tabaci*, the virus translocates from the alimentary gut to the salivary glands via the hemolymph, without apparent replication. The virus was detected in a small proportion of the F1 progeny of viruliferous females, but the F1 progeny was unable to cause viral infection of test plants. Therefore, transovarial transmission is not evidenced for TYLCTHV.

## Figures and Tables

**Table 1:** TYLCTHV-infected organs and hemocytes of *B. tabaci* detected by immunofluorescence assay.

Organ	*n*	Infection Rate (%)
Midgut	12	100
Gastric caecum	12	100
Filter chamber	12	100
Hindgut	12	0
Primary salivary gland	12	100
Ovariole	12	100
Hemocyte	12	0

**Table 2:** TYLCTHV-infected organs and hemolymph of *B. tabaci* detected by PCR.

AAP	*n*	Infection Rate (%)		
		Midgut	Hemolymph	Primary Salivary Gland
2 h	10	0	0	0
3 h	10	20	10	0
4 h	10	50	10	0
14 h	10	100	70	80
24 h	10	100	80	70

AAP: acquisition access period.

## Data Availability

The data presented in this study are available in supplementary table.

## Supplementary Materials

The following are available online at https://www.mdpi.com/2075-4450/12/2/181/s1, Table S1: Relative amount of virus.
